# Osteochondrosis in horses: An overview of genetic and other factors

**DOI:** 10.1111/evj.14518

**Published:** 2025-04-29

**Authors:** Lola Martinez‐Saez, Pablo J. Marín‐García, M. Lola Llobat

**Affiliations:** ^1^ Molecular Mechanisms of Zoonotic Diseases (MMOPS) Research Group, Departamento Producción y Sanidad Animal, Salud Pública y Ciencia y Tecnología de los Alimentos (PASAPTA), Facultad de Veterinaria Universidad Cardenal Herrera‐CEU, CEU Universities Valencia Spain; ^2^ Departamento Producción y Sanidad Animal, Salud Pública y Ciencia y Tecnología de los Alimentos (PASAPTA), Facultad de Veterinaria Universidad Cardenal Herrera‐CEU, CEU Universities Valencia Spain

**Keywords:** breeds, genetic basis, horse, osteochondrosis, prevalence

## Abstract

Osteochondrosis (OC) is a frequent manifestation of developmental orthopaedic disease, and its severe clinical presentation is known as OC dissecans (OCD). OC is defined as a disruption of the endochondral ossification process in the epiphyseal cartilage, and this disease has been reported in different mammalian species, including humans, dogs, pigs, and horses. OCD is an important cause of lameness in sport horses and is a common cause of impaired orthopaedic potential, whose clinical signs may be of minimal magnitude or manifest as severe joint effusion or clinically noticeable lameness. The aetiology of OCD is unknown, although it has traditionally been considered to be multifactorial. In addition to genetic factors, associated factors include both non‐genetic elements such as rapid growth, nutrition, trauma, anatomical conformation, and biomechanics. Since the prevalence of the disease varies greatly depending on the horse breed, from 13% in Swedish Warmblood to 53% in Lusitano breed, genetic factors have a great relevance in the appearance and development of OCD in horses. Many genetic modifications have been related, and the genes involved can be grouped into five clusters, related to fundamental functions for the correct development and regeneration of cartilage, such as collagen, laminin, cell signalling, matrix turnover, and transcriptional regulation. Changes in genes such as *COL3A1*, *COL5A1*, *COL5A2*, *COL24A1*, *COL27A1* (collagen cluster), *LAMB1* (laminin cluster), *PTH*, PHT receptors, and *IHH* (cell signalling), and genes encoding matrix metalloproteinases have been related to the occurrence and severity of diseases in different equine breeds. This review summarises the main factors associated with OC in horses, with particular emphasis on genetic factors.

## 1 INTRODUCTION

Osteochondrosis (OC) is a frequent manifestation of developmental orthopaedic disease and when OC occurs with greater severity it is known as OC dissecans (OCD). OCD has been described as the appearance of the osteochondral fragments in the joints.^1^ It can be defined as a disruption of the endochondral ossification process in the epiphyseal cartilage^2^ with (osteochondritis) or without (osteochondrosis) inflammation.^3^ This disease is not species specific and can affect several species of mammals, including human,^4^ dog,^5^ pig,^6^ cattle,^7^ turkey,^8^ chicken,^9^ and horse.^10^ Symptoms in human patients with OCD change depending on the stage or location.^4^ In dogs, clinical signs are quite like those in humans, with the most common being continuous or intermittent lameness, pain during movement, and abnormal posture of the affected limb.^11^ In pigs, OC usually presents without clinical signs, or with mild signs, such as lameness, atypical gait, and reluctance to stand or move.^12^

**FIGURE 1:**
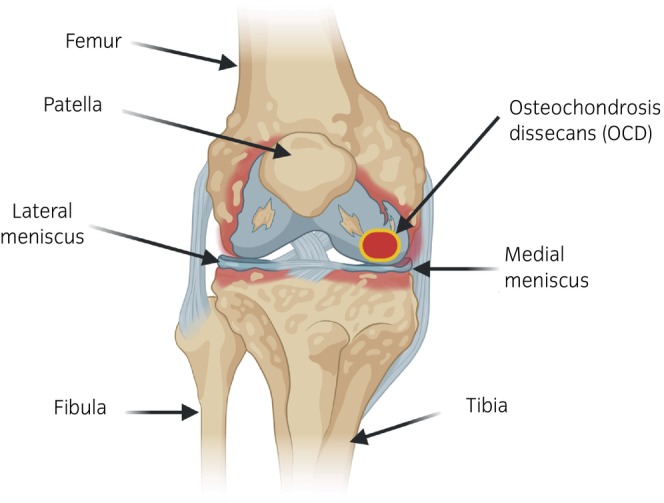
Representation of osteochondrosis dissecans (OCD) lesion in the equine stifle.

**FIGURE 2:**
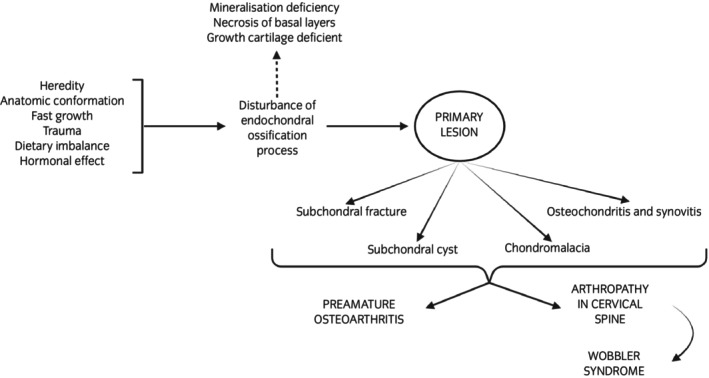
Associated factors involved in the pathogenesis of osteochondrosis dissecans (OCD) and schematic representation of the development of lesions in horses.

In sport horses, OCD is a common cause of lameness.^13^ The clinical signs can range from non‐painful, slight joint effusion, which is the most common, to more severe clinical signs that present with pain such as lameness, and this variation complicates the characterisation and diagnosis of this pathology.^14^ This disease can appear in various sites including the femoropatellar, metacarpophalangeal, metatarsophalangeal, or tarsocrural joints, depending on the equine breed, among other factors.^15^ The morphology of the lesions does not reveal the cause of the disease. Two forms of OC have been proposed: an idiopathic form, where the defect is inherent to the cartilage, and an acquired form, resulting from biomechanical, nutritional, and metabolic influences on normal cartilage.^16^ However, this classification is outdated, since if proteins are involved in pathological processes, genetics is also involved. The control of the amount, timing, and location of the proteins involved is controlled by genetic regulators. OCD results from a failure of the process by which growth cartilage is replaced by bone tissue, also known as the endochondral ossification process.^17^ Growth cartilage is found in the metaphyseal growth plate (metaphysis) and in the epiphyseal growth cartilage, which is found immediately behind the articular cartilage of immature joints.^3^ It is in this growth cartilage where an alteration in the development of the chondrocytes occurs. This phenomenon gives rise to primary lesions of the cartilage that can lead to other secondary lesions such as fragmentation, thus giving rise to OCD.^18^ The separated fragment, also known as an osteochondral lesion (Figure 1), may remain in place or become loose within the joint, causing premature osteoarthritis (OA) or arthropathy and in the cervical spine, potentially resulting in cervical vertebral stenotic myelopathy (Figure 2).^3^, ^19^, ^20^ OCD has great economic impact not only due to the veterinary and treatment costs, but also due to long‐term management, loss of animals' value, and the reduction of sporting performance due to the associated clinical signs and the high probability of subsequent complications.^10^ This disease is considered a multifactorial disorder, since there is no single aetiological factor that can explain it on its own.^3^ This review summarises the aetiology and the factors related to OCD in horses, focusing mainly on the genetic factors associated with the appearance and development of disease.

## 2 EPIDEMIOLOGY OF OCD IN HORSES

This disease is one of the most common orthopaedic conditions, affecting 20%–25% of foals. It impacts over 20,000 foals annually in Northern Europe, with the hock and fetlocks being the most affected joints, although prevalence varies by age and equine breed.^21^

Inasmuch as this disease is a disruption of the endochondral ossification process, and this process is typically only active during the 7 months after birth, OC lesions will usually develop during this period,^22^ with the youngest foal presenting dissecting lesions at around 3 days of age.^16^ However, many horses do not develop clinical signs associated with the disease until they start training and require greater athletic performance.^23^ Moreover, the OC timeline seems slightly different in different joints and breeds.^23^, ^24^, ^25^, ^26^ For example, tarsocrural joint lesions often do not become clinically apparent until later in life when training begins.^27^ In the Lusitano breed, a prevalence of 31% was found in this region in horses ranged between 1 and 12 years^28^ and different studies found lesions in foals aged between 6 and 18 months of different breeds,^29^ including French Trotter Standardbreds^30^ and Thoroughbreds.^31^ The variation in the age of onset of clinical signs in the OCD could be due to the involvement of other factors such as diet and housing.^29^

The prevalence of this orthopaedic disease varies greatly depending on the horse breed, and ranges between 13% and 53.3% in Swedish Warmblood and Lusitano breeds, respectively (Table 1). These data suggest that one of the most relevant factors in the development of OCD is the genetic background of the horse.

**TABLE 1:** Prevalence and most affected joint of osteochondrosis (OC) or osteochondrosis dissecans (OCD) depending on the equine breed.

Equine breed	OC(D) prevalence (%)	Most affected joint	References
Norwegian Standardbreed	50.7%	Distal intermediate ridge of the tibia and lateral trochlear ridge of the talus	[166]
Lusitano	53.3%	Distal intermediate ridge of the tibia in the tarsocrural joint	[28]
Hanoverian Warmblood	14.3%	Hock joints	[167]
South German Coldblood	OC: 61.7% OCD: 26.9%	Dorsal aspect of the sagittal ridge of the third metacarpal or metatarsal bone	[168]
Swedish Warmblood	13.0%	Fetlock	[169]
Dutch Warmblood	44.3%	Tarsocrural joints	[170]
Spanish Purebred	48.8%	Tarsocrural joints	[171]
French Sport	13.3%	Forelimb fetlocks	[172]
Zangersheide	15%		[173]
Cheval de Selle Luxembourgeois	5%		
Koninklijk Warmblood	1%		
Hanoverian	10%		
Belgian Warmblood	15%		
Cheval de Sport Belge	35%		
Anglo European Studbook	5%		
Selle‐Français	8%		
Thoroughbred	92%	Tarsal joint	[174]

## 3 NON‐GENETIC FACTORS RELATED TO OCD

OCD is a multifactorial aetiology disorder (Figure 2) and the most cited non‐genetic factors are fast growth, nutrition, trauma and anatomic shape.^3^ The results regarding the role of rapid growth in the development of OCD are contradictory between pigs, dogs and horses. Thus, some large population analyses in pigs indicated a positive genetic correlation between rapid growth and prevalence of OC,^32^, ^33^ whereas other studies demonstrated that the development of OCD lesions was not decreased by reducing growth rate.^34^, ^35^ Similarly, studies performed in dogs show contradictory conclusions. The main study that found an association between rapid growth and the development of OCD lesions was carried out in the Great Dane canine breed.^36^ In horses, the morphology of the initial lesions of articular OC, and the fact that the lesions occur focally in specific sites and are not generalised, contradict theories published by Jeffcott and Henson^16^ and Shingleton et al.,^37^ who proposed that an increase in circulating insulin can cause hypertrophy in the chondrocytes surrounding the cartilage canals. Thus, according to the studies published to date, there is no evidence that rapid growth is a determining factor in the development of equine OCD. Excess or deficiency in some minerals in the diet, such as calcium, phosphorus, copper, zinc, or vitamins A, C, D, and biotin, have been proposed as factors related to OCD in some species. However, diets supplementing these biomolecules have not demonstrated this relationship in pigs,^38^ dogs,^39^ or horses.^40^

Although some studies in humans have shown that trauma can cause osteochondral fractures, the role of trauma in the initial development of the disease is limited. Most cases in animals and humans do not include traumatic events, and severe trauma alone does not explain the specificity of the lesions and the fact that they are usually bilaterally symmetrical.^3^, ^41^, ^42^, ^43^ These results indicate that, although the role of trauma may depend on the stage of the disease, it is not a factor with a relevant role in the pathogenesis of the disease. The effect of joint conformation on the development of OCD has been demonstrated in different species. Specifically, in humans, it has been observed that the anatomical shape and function of the knee joint determine the appearance of OCD.^44^, ^45^ In pigs, an overload of the joints due to their anatomical shape seems to be the most important cause of disease.^46^, ^47^ But, as has been demonstrated in pigs,^48^ anatomical modifications are closely related to the genetic background; therefore, we could assume that the most influential factors in the appearance and development of OCD are genetic factors and the molecular background.^3^

Several authors have linked biomechanical influences with the appearance and severity of OC(D). For the transformation of cartilage into bone, it is necessary that this cartilage is irrigated by blood vessels, and excessive loading during the first months of a foal's life can produce necrosis, hindering this ossification process.^34^, ^35^ Osteochondral lesions attributed to biomechanical influences tend to be focal lesions due to the variation in the distribution of blood vessels in different areas of the cartilage and changes in the arrangement of the components of the cartilage matrix, making some regions more sensitive to necrosis.^6^, ^37^, ^38^ Nevertheless, it is recommended that foals perform controlled pasture exercise because moderate physical activity has several benefits on the cartilage structure, such as greater GAG content, lower levels of fragments of degraded type I collagen, or greater percentage of viable chondrocytes.^39^, ^40^, ^41^, ^42^

## 4 GENETIC FACTORS RELATED TO OCD

Although the genetic basis of this disease remains unclear, several studies have completed genome‐wide association studies (GWAS) to identify candidate genes responsible for OC in different equine breeds.^43^ Genome‐wide significant quantitative trait loci (QTLs) for OC have been identified in Hanoverian Warmblood horses, and comparing the equine QTL positions with the conserved chromosomal candidate region(s) between humans and horses, several candidate genes involved in cartilage maturation have been demonstrated.^49^, ^50^ In the same breed, Lampe et al. also identified candidate genes responsible for the disease.^51^, ^52^, ^53^ Additional candidate genes for OCD have been identified in South German Coldblood horses,^54^, ^55^, ^56^ in Thoroughbred horses,^57^ and in Norwegian trotters.^58^ A large number of genes appear to be related to the occurrence of OCD in horses, and most of them can be grouped into five main clusters related to fundamental functions for the correct development and regeneration of cartilage, such as collagen, laminin, cell signalling, matrix turnover, and posttranslational modifications.^43^

### 4.1 Collagen cluster

Collagen is one of the most abundant proteins in the extracellular matrix, and mutations in genes encoding various types of collagens can lead to several skeletal diseases. Mutations can alter the development, conformation, and function of different collagen fibres.^59^ Specifically, mutations or changes in the expression of genes encoding collagen type 3 have been related to OA. For example, an increase in gene expression levels of collagen type 3 alpha 1 (*COL3A1*) has been observed in chondrocytes at the early stages, while it decreases toward the end of the disease. One of the functions of this gene is to reduce the inflammatory process of OA. To do this, it reduces the cluster of differentiation 4 (CD4) memory T cells and natural killer (NK) cells, thus inhibiting the immune response.^60^ Recently, a study carried out in Thoroughbred horses showed that a single nucleotide polymorphism (SNP) in this gene is closely related to the possibility of fracture in individuals who carry it.^61^

Collagen type 5 is found in tissues containing type 1 (*COL5A1*) collagen and acts to regulate the assembly of heterotypic fibres composed of both type 1 and 5 collagen.^62^ The *COL5A1* gene is contained in equine chromosome (ECA) 25, around the QTL for OCD of the fetlock, and is associated with epiphyseal dysplasia.^55^ It has also been associated with human OA.^63^ The collagen type 5 alpha 2 (*COL5A2*) protein is related to the structural components of cartilage,^64^ and some authors have linked mutations of this gene with Ehler–Danlos syndrome, which alters the natural properties of connective tissue, making it too elastic.^65^, ^66^ In horses of the Polish Warmblood breed, a mutation of *COL5A2* has been related to the presence of OC(D) in the two joints that make up the stifle of these carrier animals.^67^ The collagen type 27 alpha 1 (*COL27A1*) gene, as *COL5A1*, is contained in equine chromosome 25, around the QTL for OCD on the fetlock, and is also associated with epiphyseal dysplasia.^57^ *COL27A1* is another gene that could be related to the appearance of OC(D), since it is mainly found in growth cartilage.^68^ In a GWAS study, mutations in this gene were shown to be related to knee OA in human patients,^69^ and other studies suggest that it could be useful in the early detection of this disease, since its expression increases when a lesion appears.^70^ Collagen type 24 alpha 1 (*COL24A1*) was also identified as a candidate gene for OC(D) in Hanoverian Warmblood horses because of its role in bone formation, since when this process fails is when the pathology appears.^53^ Also, Sevane et al.^71^ identified a non‐synonymous *COL24A1* polymorphism as being suggestively associated with locomotion in Spanish purebred horses, an equine breed with a high prevalence of OC(D). This gene, related to embryonic bone formation and type 1 collagen fibrinogenesis, has been found to be involved in the transcription process of trabecular bone and periosteum in studies carried out in mice.^72^ Table 2 lists collagen genes related to equine OC, all related to extracellular matrix organisation as a biological process (Panther software). Considering these studies together suggests genes encoding different types of collagens could play a relevant role in the development of OCD in horses, so further larger studies in other equines would be valuable.

**TABLE 2:** Collagen cluster genes related to equine osteochondrosis (OC), equine breed where it is found, chromosome number, description and function.

Gene	Equine SNP location	Equine breed	Full name and description	References
*COL3A1*	Chromosome 18	Thoroughbred	Collagen, type III, alpha 1. Collagen type III occurs in most soft connective tissues along with type I collagen (1466 aa)	[57]
*COL5A1*	Chromosome 25	South German Coldblood	Collagen, type V, alpha 2. Type V collagen is a member of group I collagen (fibrillar forming collagen). It is a minor connective tissue component of nearly ubiquitous distribution. Type V collagen binds to heparan sulphate, thrombospondin, heparin, and insulin. Type V collagen is a key determinant in the assembly of tissue‐specific matrices (1499 aa)	[54]
*COL5A2*	Chromosome 18	Polish sport breeds	Collagen, type V, alpha 2. Type V collagen is a member of group I collagen (fibrillar forming collagen). It is a minor connective tissue component of nearly ubiquitous distribution. Type V collagen binds to heparan sulphate, thrombospondin, heparin, and insulin. Type V collagen is a key determinant in the assembly of tissue‐specific matrices (1499 aa)	[67]
*COL24A1*	Chromosome 5	Spanish purebred Hannoverian Warmblood	Collagen, type XXIV, alpha 1. Participates in regulating type I collagen fibrillogenesis during foetal development (1714 aa)	[71] [53]
*COL27A1*	Chromosome 25	South German Coldblood	Collagen, type XXVII, alpha 1. Plays a role during the calcification of cartilage and the transition of cartilage to bone (1860 aa)	[54]

### 4.2 Laminin cluster

Within the extracellular matrix, laminins are one of the main non‐collagenous components. This family of glycoproteins is related to various cellular processes such as adhesion, differentiation, and migration, in addition to cancer and growth of neurological components. These functions depend on the connections between these gycoproteins.^73^ Different laminin isoforms of these glycoproteins exist and are differentially distributed during development and in tissues.^74^

Different genes are associated with OC(D) and other pathologies (Table 3). Related to laminin cluster, an isoform of laminin which has been identified in human adult articular cartilage is Laminin‐1 (*LAMB1*).^75^ This glycoprotein, together with collagen type 4, mainly constitutes the pericellular matrix (PCM) of healthy cartilage tissues, but in damaged cartilage only collagen type 4 is found, not laminin.^76^ In this way, some studies suggest that alterations in PCM could be related to OA due to changes that occur around chondrocytes.^77^, ^78^ Although laminins play a fundamental role in extracellular matrix formation of cartilage and PCM homeostasis, only one study relates the laminin 1 isoform to OC in horses. This study is a GWAS study carried out in 629 foals of Hanoverian Warmblood horses that found different markers linked to OC, including a QTL that would be homologous to the human *LAMB1* gene as a candidate gene.^49^ Biological functions of this gene are varied and related to substrate adhesion‐dependent cell spreading, cell migration, tissue development, axon guidance, cellular component assembly, animal organ morphogenesis and extracellular matrix organisation, as collagen cluster genes (Panther software).

**TABLE 3:** Genes linked to OC(D) and genes involved in other joint pathologies.

Gene/protein^a^	Pathologies associated	References
*COL3A1*	OA	[60]
	Fractures	[61]
*COL5A1*	Epiphyseal dysplasia	[55]
	OA	[63]
*COL5A2*	Ehler–Danlos	[65, 66]
	OC(D)	[67]
*COL27A1*	Epiphyseal dysplasia	[57]
	OA	[68]
*COL24A1*	OC(D)	[53]
LAMB1	OA	[77]
*LAMB1*	OC	[49]
*PTH2R*	OC	[57]
	OA	[95]
*IHH*	OC	[96, 97, 98, 99]
*Wnt*	OA	[102, 103]
	OC	[104, 105]
CGRP	OA	[109]
	Migraine	[110, 112]
RCP9	POF	[42]
*FRZB*	OA	[115]
MMP‐13, *MMP‐13*	OC	[120]
*MMP‐13*	OA	[123]
*Runx2*	OC	[123]
*MATN1*	OA	[131]
*HYAL1*	Mucopolysaccharidosis type	[133]
	IX OC(D)	[67]
*UGDH*	OC(D)	[49]
	OA	[136]
SCL35D1	OC(D)	[138, 139]
*NCDN*	OC(D)	[50]
*TLK2*	OC	[139]
*AOAH*	OC(D)	[54]
IL6	OA	[149, 150]
	OC(D)	[151]
	Rheumatoid arthritis	[153]
*TBC1D22A*	OA	[155]
LGALS3	Equine post‐traumatic arthritis	[160]
	Juvenile idiopathic arthritis	[160]
	Rheumatoid arthritis	[161]
	Antigen‐induced arthritis	[161]
*SGK1*	OC(D)	[162]
*XIRP2*	OC(D)	[51]
GALNT13	OC(D)	[54]
*CLCA4*	OC(D)	[51]

Abbreviations: OA, osteoarthritis; OC(D), osteochondritis (dissecans); POF, palmar/plantar osseous fragments.

^a^ Genes are written in italics while proteins are written in non‐italic font.

### 4.3 Cell signalling cluster

Concentrations of vitamin D (1,25(OH)_2_D) and serum calcium regulate the secretion of one of the principal calciotropic hormones, parathyroid hormone (PTH).^66^, ^79^ This hormone is also anabolic for osteocytes^67^ and chondrocytes^68^ and is capable of promoting the repair of damaged cartilage and bone in injuries similar to those of OC(D),^80^ so genes related to this hormone could also be candidates responsible for this disease.

In humans, defects in PTH receptor are known to be the cause of Jansen's metaphyseal chondrodysplasia, Blomstrand chondrodysplasia, or enchondromatosis, among other diseases.^81^, ^82^, ^83^, ^84^, ^85^, ^86^, ^87^ In Lusitano horses, PTH concentrations decreased with age and increased in horses diagnosed with OC.^88^ Similar results were found by van Oldruitenborgh‐Oosterban et al. (1999)^89^ indicating that increased concentrations of PTH and 1,25(OH)2D in plasma do not modify concentrations of ionised calcium, even though one of the main functions of PTH is to maintain calcium concentration, so the relationship between increased PTH concentrations and the appearance of the disease is related to other factors.^89^

**FIGURE 3:**
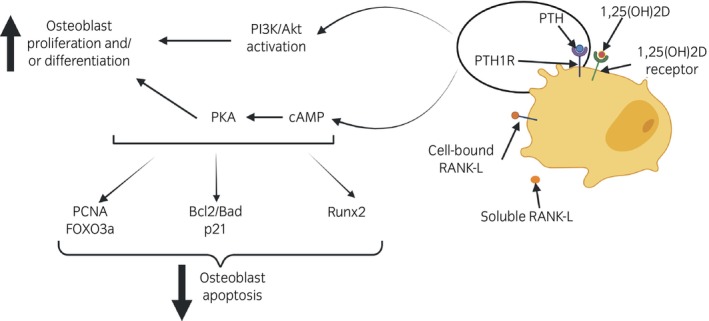
Schematic representation of parathyroid hormone pathways involved in activation of osteoblast proliferation, differentiation, and apoptosis. 1,25(OH)2D, vitamin D; Bcl2/Bad, B‐cell lymphoma 2 associated agonist of cell death; cAMP, cyclic adenosine monophosphate; FOXO3a, transcription factor Forkhead box protein O3; PCNA, proliferating cell nuclear antigen; PI3K/Akt, phosphatidylinositol 3‐kinase (PI3K)/protein kinase B; PKA, protein kinase A; PTH, parathyroid hormone; PTHR1, parathyroid hormone receptor 1; RANK‐L, receptor activator of nuclear factor kappa‐Β ligand; Runx2, Runt related transcription factor 2.

Three receptors for PTH have been described: parathyroid hormone receptor 1 (PTH1R), parathyroid hormone receptor 2 (PTH2R) and parathyroid hormone receptor 3 (PTH3R), although only the first two have been found in mammals. PTH1R is downregulated by PTH in osteoblastic cells and is thought to be involved in PTH action in bones.^90^ The union between PTH and its receptor PTH1R activates bone formation and stimulates the maturation of osteoclasts, increasing bone resorption.^91^ The cell membrane of osteoblasts expresses the PTH1R in addition to receptors for 1,25(OH)_2_D.^92^, ^93^ An increase in PTH concentrations stimulates the release of the receptor activator of nuclear factor kb ligand (RANKL), which, by binding to its receptor, activates the formation of osteoclasts (Figure 3).^94^

PTH2R has a much more specific distribution in tissues compared with PTH1R and only PTH can activate it, unlike PTH1R, which can also be activated by PTH‐like hormone. Due to the important role that parathyroid hormone plays in regulating calcium metabolism, some studies relate this gene to OC due to the large percentage that this mineral represents in bone composition. In fact, Meulenbelt et al. conducted a study in humans with early‐onset OA where they observed a possible relationship between this disease and a variant in the *PTH2R* gene.^95^

Indian Hedgehog (*IHH*) is another gene involved in the cartilage ossification process, acting specifically on chondrocytes in their differentiation, proliferation, and maturation.^96^ *IHH* is mainly related to the regulation of gene expression (Panther software). It has been shown that this gene is regulated by negative feedback from parathyroid hormone‐related protein (*PTHrP*) that controls chondrocyte differentiation.^97^ Some studies show that in the cartilage of horses with OC there is greater expression of *IHH* and *PTHrP*, among other genes, especially in the deeper layers and in the most advanced lesions of the disease, thus demonstrating the important role that this gene plays in the development of OC.^98^, ^99^ A study in Belgian Warmblood horses comparing a group of horses with OC with a healthy group determined that the expression of some genes was altered in those horses affected by OC, thus altering some metabolic processes related to OC such as cartilage formation or repair.^96^

Wnt is a protein family involved in cellular development and maintenance processes.^100^ Wnt/β‐catenin genes help the proper functioning of cartilage‐related functions such as the endochondral ossification process, cartilage delimitation and the correct structure of the growth plate through the control of chondrocyte maturation, function and phenotype.^96^, ^101^ Wnt is related to diseases in which there is cartilage degeneration such as OA or OC, since when it is deregulated by an increase in β‐catenin it promotes cartilage loss.^102^, ^103^ In horses, some studies have shown that those individuals with OC show increased expression of some canonical and non‐canonical Wnt pathways genes than healthy individuals.^104^ Moreover, recent studies have demonstrated that at the margins of the OC lesions there is a decrease in β‐ catenin concentrations and an increase in an inhibitory component of the canonical Wnt pathway that inhibits this pathway.^105^

Bone repair is a complex process that involves several molecules, including calcitonin and calcitonin gene‐related peptide (CGRP), a sensory neuropeptide involved in painful pathologies such as migraines due to its relationship with pain and neurogenic inflammation.^106^ In joints, this neuropeptide protects healthy chondrocytes and contributes to their anabolism,^107^ promoting osteogenesis and bone remodelling.^108^ Recent studies carried out in humans have demonstrated the therapeutic effect of the administration of anti‐CGRP antibodies and CGRP agonists for the treatment of pain due to reduction in neurogenic inflammation and in signalling of the trigeminovascular pathway,^109^ suggesting that *CGRP* may also be a therapeutic target for the control of OA and other pathologies, such as chronic migraine.^110^, ^111^, ^112^ The gene encoding this peptide receptor, named calcitonin gene‐related peptide receptor component protein 9 (*RCP9*), also has an anabolic activity for chondrocytes, and it is part of a QTL related to the presence of plantar/palmar osseous fragments in fetlocks.^42^

Secreted frizzled‐related protein (FRZB) has been observed in various forms of OA.^113^ It is involved in skeletal development and can be found in different bones such as the craniofacial bones, the ribs or those that constitute the appendicular skeleton, as well as in the articular chondrocytes of the cartilage.^114^ Functional mutations in *FRZB* in women attribute greater susceptibility for OA,^115^ and knockout mice of the *FRZB* gene have cartilage damage by increasing the expression and activity of matrix metalloproteinases.^116^ In horses, this gene is related to canonical and non‐canonical Wnt signalling pathways, but the studies of this gene in horses are scarce and further research is needed to address the function of this gene in equines and its potential role in OC(D).

### 4.4 Matrix turnover cluster

Matrix metalloproteinases (MMPs) are a family of 28 zinc‐dependent endopeptidases involved in the degradation of various proteins in the ECM, including collagen and laminin.^117^ Most MMPs are related to pathological events and inflammatory processes, and MMP‐13 has been directly related to OC in different equine breeds.^118^ During embryonic development, the *MMP‐13* gene is manifested in the skeleton, and its expression increases in synovial cells and chondrocytes when diseases such as OA appear. When this metalloproteinase disappears, cartilage problems appear, such as a deregulation of collagenases that causes an accumulation of collagen in the interstitium, hypertrophy of the growth plate, and delays in the creation and vascularisation of the endochondral ossification centres and in the ossification process itself.^119^ *MMP‐13* expression is notably increased in OC lesions compared with healthy joints in horses.^120^

The main regulatory factor of *MMP‐13* transcription is Runt related transcription factor 2 (*Runx2*). *Runx2* is a transcription factor (Figure 3) of the Runx family,^121^ which is mainly expressed in osteoblasts and terminal hypertrophic chondrocytes in growth plates^122^ and related to biological processes such as cartilage development or ossification, among others. Increased Runx2 and collagen type I (COL1) in these lesions may be responsible for the high expression of the *MMP‐13* gene. In vitro studies have shown that this metalloproteinase degrades aggrecan and collagen, and its concentrations increase in chondrocytes affected by OCD.^123^, ^124^ In horses with OC, cartilage lesions exhibit higher expression of Runx2 compared with healthy cartilage in the same joint.^123^ The expression of this gene in chondrocytes produces mineralisation of the cartilage matrix, alkaline phosphatase activity, and expression of genes typical of chondrocyte maturation, including type 10 collagen (*COL10*) and *MMP‐13*, which also explains the increase in expression of these genes when there are OC lesions.^120^, ^124^, ^125^ Studies carried out in mice have shown that in those *Runx2* ^−/−^ individuals there is no expression of vascular endothelial growth factor (VEGF), an angiogenic factor, so hypertrophic cartilage does not become vascularised during the endochondral ossification process.^126^ Therefore, this factor, together with MMP, could also be involved in other pathological processes with inflammation, such as OC.

In addition to collagen and laminin, other components are present in the ECM, including protein matrilin 1 (MATN1) or hyaluronan (HA). MATN1 is a member of the matrilin protein family; in fact, it was the first one to be identified.^127^ Although it is expressed in several skeletal tissues, it is mainly found in hyaline cartilage.^128^ MATN1 is involved in the structuring of multiple molecular aggregates of the extracellular matrix of cartilage^129^, ^130^ and studies carried out in mice have shown that those MATN1 deficient individuals, when faced with mechanical challenges in the joints, have greater cartilage degeneration than normal individuals, suggesting that this gene could be a target in the prevention of degenerative diseases such as OA.^131^ *MATN1* gene mutations have been associated with a variety of inherited chondrodysplasias.^132^ It is an interesting candidate for future study in equine OC.

Within articular cartilage, one of the major components of the extracellular matrix is hyaluronic acid or hyaluronan. This glycosaminoglycan is essential due to its multiple functions, among which joint lubrication stands out, helping in movement and hydrodynamics, and in cell differentiation, migration, and proliferation.^133^, ^134^ This glycosaminoglycan is broken down by the action of the enzyme hyaluronidase.^120^ Hyaluronidase (*HYAL*) gene family members have been described as candidate genes for OC, Specifically *HYAL1*, *HYAL2*, and *HYAL3*, with HYAL1 encoding a lysosomal hyaluronidase and HYAL2 and HYAL3 encoding two proteins like hyaluronidase. These genes are similar but do not perform the same function. In humans, mutations in *HYAL1* have been associated with mucopolysaccharidosis type IX.^133^, ^134^ In Polish Warmblood horses, a mutation in the *HYAL1* gene is related to clinical signs compatible with OC(D) both before and after being subjected to athletic tests.^67^

### 4.5 Posttranslational modifications

Different genes involved in protein synthesis, transcription regulation, and posttranslational modifications may influence the development of OC. Studies carried out in Thoroughbred horses revealed a SNP on chromosome 3 significantly associated with OCD. Examining the region 1 Mb on each side of the SNP, the authors found the UDP‐glucose dehydrogenase (*UGDH*) gene, which is necessary for the production of glycosaminoglycans in the extracellular matrix.^135^ UGDH transforms UDP‐glucose into UDP‐GlcUA, which is necessary for HA synthesis. The knockdown of *UGDH* reduces UDP‐GlcUA concentrations with a corresponding decrease of HA,^131^ which produces a deregulation of cartilage homeostasis that influences the appearance and development of diseases such as OA.^136^

Solute carrier family 35 member D1 (*SCL35D1*), also known as chondroitin sulphate (CS), is a glycosaminoglycan distributed in the cartilage.^36^ An epitope called CS‐846 has been measured in a few species, including the horse. This epitope, after separating from the aggrecan protein in the extracellular matrix, is released into the synovial fluid,^137^ and some studies suggest that concentrations of this epitope are more decreased in the synovial fluid of affected horses between 24 and 48 months old in comparison with affected horses between 9 to 23 months old.^138^, ^139^ CS has been used as a biomarker of cartilage matrix degeneration and degree of joint damage in some individuals suffering from OCD.^3^ Although some studies have related CS with OCD in horses, neither the severity scores of the macroscopic lesions nor the number of lesions are significantly correlated with this biomarker, but it is significantly correlated with lesion count and total radiographic scores and could be a biomarker for OCD in horses.^140^, ^141^ This could indicate an increase in the synthesis of cartilaginous aggrecan during the development of the disease. Therefore, it has been concluded that it is closely linked to OC(D).^3^, ^141^

Calcium‐/calmodulin‐dependent protein kinase II phosphorylation is downregulated by the neurochondrin (*NCDN*) gene and is expressed in bone and cartilage cells such as osteocytes, osteoblasts, and chondrocytes.^141^ Mochizuki et al. carried out a study in mice with which they were able to demonstrate the role of the *NCDN* gene in various cellular processes of chondrocytes such as proliferation or differentiation, as well as their survival.^142^ In their research, mice with mutations of this gene had an alteration in the transformation of cartilaginous tissue to bone tissue. A study carried out in Hanoverian horses has indicated this gene as a candidate for OC(D) involvement due to the associations between this disease and an SNP associated with the *NCDN* gene.^50^

Tousled‐like kinases (TLKs) are nuclear serine/threonine kinases whose function focuses on DNA replication and chromatin transcription and remodelling,^143^ so when they are upregulated, animals with OCD‐like lesions can increase cartilage growth rate and recovery due to increased cell division.^144^ In fact, Austbø et al. revealed an upregulation in two genes, *TLK2* gene and CD465746.1, in a study with cartilage from Standardbred foals considered predisposed to OC since their parents both suffered from the disease.^139^ Other transcription regulators could be related to OC, such as F‐box proteins. F‐box protein 25 (*FBXO25*) encodes a member of the F‐box protein family. F‐box proteins are related to several metabolic processes of the cell cycle, transcription, signalling, and proteolysis.^58^ Furthermore, FBXO25 has been linked to the regulation of osteogenesis, so it could be a potential target in bone defect regeneration therapies.^145^

Acyloxyacyl hydrolase (AOAH) is a lipase that acts on lipid A of lipopolysaccharides of gram‐negative bacteria. Although its role in bone metabolism remains unclear, some studies demonstrate that AOAH is involved in bone metabolism, since its decrease promotes osteoclast differentiation and improves bone resorption by marrow‐derived macrophages.^146^ A study has linked OCD in fetlocks with three SNPs located in different noncoding DNA sequences of this gene in South German Coldblood horses.^54^ Both the *AOAH* gene and the interleukin 6 (*IL6*) gene are located on equine chromosome 4 (ECA4) and both genes are related to the presence of bone fragments in both the fetlock and the plantar area.^42^ IL‐6 is a proinflammatory cytokine which stimulates the production of osteoclasts and bone replacement.^147^, ^148^ Some studies have demonstrated that OA positive horses show higher expression of IL‐6 in the synovial fluid than OCD or healthy horses, suggesting that IL‐6 is involved in the physiopathology of this disease.^149^, ^150^ However, the role of IL‐6 in OC(D) is controversial. Stimulation of production of B and T lymphocytes by this cytokine generates a prolonged inflammatory response over time, having demonstrated an improvement in rheumatoid arthritis patients treated with anti‐IL‐6 receptor antibodies.^151^ However, a study carried out in mice has shown that IL‐6 deficiency reduces the production of chondrocytes proteoglycans and increases their degradation, producing a cartilage loss, thus suggesting that this interleukin could have a protective role in diseases that involve cartilage degeneration.^152^ In horses with carpal joint disease, increased concentrations of IL‐6 were detected in the synovial fluid in those joints with chip fractures and measurements of this marker in synovial fluid could be a way to detect osteochondral fragmentation.^153^

TBC1 domain family member 22A gene (*TBC1D22A*) encodes a protein that regulates small GTPases, essential for a large number of functions such as cellular movement, differentiation or growth, as well as signal transduction and fat transport.^154^ In a study where chondrocytes were stimulated with different interleukins to examine the signalling of these small GTPases, it has been seen that a change occurs in the morphology and phenotype of these chondrocytes, which can provide information about the morphological and phenotypic changes that occur in these cells in degenerative processes such as OA.^155^

Galectins are proteins whose activity consists of regulating cellular processes such as adhesion, growth, or cell death of chondrocytes and synovial fibroblasts, among other types of cells.^156^ These proteins are found in the synovial fluid of healthy joints in horses, but their concentrations increase when there are osteochondral lesions^157^ such as those that occur in human diseases like juvenile idiopathic arthritis, rheumatoid arthritis, or antigen‐induced arthritis.^158^, ^159^ Specifically, galectin‐3 (LGALS3) is involved in inflammatory processes and bone destruction in joints and promoting the activation of synovial fibroblasts.^160^ However, its role in these diseases is controversial, and it has been suggested that LGALS3 plays a role in joint lubrication, which may prevent cartilage degeneration.^161^

LOC100073151 is a protein‐coding gene like serum/glucocorticoid regulated kinase 1 (*SGK1*). *SGK1*, in the homologous region of human DNA, is involved in a wide variety of brain and kidney processes, but it could also be closely related to OC(D) due to its role in the response to cellular stress and ischaemic necrosis.^162^ *SGK1* has also been identified as being associated with locomotor attributes in Spanish purebred horses.^76^

Xin actin‐binding repeat containing 2 (*XIRP2*) has been identified as a candidate gene for OC(D) in 2009.^51^ This gene is a composition of 28 Xin proteins, which are natural proteins of striated muscle.^163^ One of these proteins is the polypeptide *N*‐acetylgalactosaminyltransferase 13 (GALNT13). In the study carried out in South German Coldblood horses, those individuals with mutations in two SNPs of this gene were more susceptible to developing OC(D) in the hock and fetlock joints.^54^ A relationship between one of these two mutations and the presentation of clinical signs of OC(D) has also been observed in the same joints in Polish Warmblood horses.^53^

Chloride channel calcium activated family member 4 gene (*CLCA4*) and OC(D) have some relationship with OC(D) development.^51^ *CLCA4* is involved in multiple functions such as neuronal and muscular excitability, olfactory signal transduction, and transport of substances at the epithelium. *CLCA4* is capable of suppressing the phosphatidylinositol 3‐kinase (PI3K)/protein kinase B (PI3K/Akt) pathway, inhibiting cell proliferation,^164^ and osteoblast proliferation and differentiation (Figure 3).

Finally, other regulatory mechanisms of transcription and gene expression could be related to OC. Epigenetic mechanisms, such as microRNAs, are potential biomarkers for OC. Changes in the expression of certain microRNAs can affect the transcription patterns of key genes involved in osteochondral ossification. For instance, the overexpression of miR‐145 reduces the expression of crucial extracellular matrix genes like *COL2A1* and aggrecan, while increasing the expression of genes such as *Runx2* and *MMP‐13*, which are associated with collagen degradation.^165^

## 5 CONCLUSIONS

OC is a pathology that affects the formation and regeneration of cartilage of great relevance in the equine industry. Although the disease has long been considered to have a multifactorial origin, it appears that genetic factors have a much greater impact on its onset and development compared with non‐genetic factors. Alterations in the sequence or expression of certain genes associated with key proteins like collagen or laminin, cellular signalling pathways including parathyroid hormone regulation, control of extracellular matrix components in cartilage, and transcriptional regulation may significantly contribute to the development of the disease. However, comprehensive studies regarding the genetic mechanisms underlying the appearance of this disease in horses are scarce. Further research is needed to explore the role of various genes and their genetic and epigenetic regulation in the development of equine OC. Understanding how this condition manifests in the early years of life will be crucial for establishing genetic selection mechanisms to reduce its prevalence.

## FUNDING INFORMATION

Universidad San Pablo CEU, grant numbers IDOC23‐01, INDI23‐35 and GIR23‐35. Lola Martínez‐Sáez is supported by a Predoctoral Contract form the Universidad Cardenal Herrera CEU.

## CONFLICT OF INTEREST STATEMENT

None of the authors of this article has a financial or personal relationship with other people or organisations that could inappropriately influence or bias the content of the article.

## AUTHOR CONTRIBUTIONS

**Lola Martinez‐Saez:** Writing – original draft. **Pablo J. Marín‐García:** Writing – review and editing; supervision. **M. Lola Llobat:** Conceptualization; investigation; funding acquisition; writing – original draft; writing – review and editing; supervision; project administration.

## DATA INTEGRITY STATEMENT

Not applicable.

## ETHICAL ANIMAL RESEARCH

Not applicable.

## INFORMED CONSENT

Not applicable.

## Data Availability

Data sharing is not applicable to this article as no new data were created or analysed in this study.
